# The role of dung beetle species in nitrous oxide emission, ammonia volatilization, and nutrient cycling

**DOI:** 10.1038/s41598-023-30523-0

**Published:** 2023-03-02

**Authors:** Carlos C. V. García, José C. B. Dubeux, Xavier Martini, Derick Conover, Erick R. S. Santos, Bruno G. C. Homem, Martin Ruiz-Moreno, Izabela A. G. da Silva, Daciele S. Abreu, Luana M. D. Queiroz, Flavia O. S. van Cleef, Mércia V. F. Santos, Giselle G. M. Fracetto

**Affiliations:** 1 Deparment of Animal Science, Federal Rural University of Pernambuco, Recife, PE Brazil; 2 University of Florida, North Florida Research and Education Center, Marianna, FL USA; 3 University of Alberta, Alberta, Canada; 4 EMBRAPA Agrobiology, Seropédica, RJ Brazil; 5 Federal University of Rondonópolis, Rondonópolis, MT Brazil; 6 University of Lavras, Lavras, MG Brazil; 7 Center for Nuclear Energy in Agriculture, University of São Paulo, Piracicaba, SP Brazil; 8 Department of Soil Science, Federal Rural University of Pernambuco, Recife, PE Brazil

**Keywords:** Environmental sciences, Ecology, Ecosystem ecology, Ecosystem services, Grassland ecology

## Abstract

This study evaluated the role of dung beetle species alone or associated under different species on nitrous oxide (N_2_O) emission, ammonia volatilization, and the performance of pearl millet [*Pennisetum glaucum* (L.)]. There were seven treatments, including two controls (soil and soil + dung without beetles), single species of *Onthophagus taurus* [Shreber, 1759] (1), *Digitonthophagus gazella* [Fabricius, 1787] (2), or *Phanaeus vindex* [MacLeay, 1819] (3); and their assemblages (1 + 2 and 1 + 2 + 3). Nitrous oxide emission was estimated for 24 days, when pearl millet was planted in sequence to assess growth, nitrogen yield (NY), and dung beetle activity. Dung beetle species presented greater N_2_O flow of dung on the 6th day (80 g N_2_O-N ha^−1^ day^−1^) compared to soil and dung (2.6 g N_2_O-N ha^−1^ day^−1^). Ammonia emissions varied with the presence of dung beetles (*P* < 0.05), and *D. gazella* had less NH_3_^−^N on days 1, 6, and 12 with averages of 2061, 1526, and 1048 g ha^−1^ day^−1^, respectively. The soil N content increased with dung + beetle application. Dung application affected pearl millet herbage accumulation (HA) regardless of dung beetle presence, and averages ranged from 5 to 8 g DM bucket^−1^. A PCA analysis was applied to analyze variation and correlation to each variable, but it indicated a low principal component explanation (less than 80%), not enough to explain the variation in findings. Despite the greater dung removal, the largest species, *P. vindex* and their species combination, need to be more studied to get a better understanding about their contribution on greenhouse gases. The presence of dung beetles prior to planting improved pearl millet production by enhancing N cycling, although assemblages with the three beetle species enhanced N losses to the environment via denitrification.

## Introduction

Livestock production may contribute to the intensification of the greenhouse effect, with cattle enteric fermentation^1^, fresh dung^2^, and rice production^3^ being major contributors to methane (CH_4_) emissions; N fertilizers^4^ and livestock excreta (urine and dung) are major contributors to nitrous oxide (N_2_O) emissions. According to Meng et al.^5^, annual greenhouse gas (GHG) emissions from N fertilizer production and usage are predicted to be 50 g N_2_O-N, while fertilizing with untreated cattle dung emit 90 g N_2_O-N.

Dung beetles have the potential to reduce GHG emissions by aerating the soil and breaking the anaerobic zones formed under the dung crust^6^, which can affect the interaction of deposited excreta with soil microbial populations^7^. Dung beetles are coprophagous insects (Insecta class and Coleoptera order) that play an important role in N cycling in both temperate and tropical agricultural grasslands^8^. They may help reduce GHG emissions and improve carbon sequestration by enhancing grass growth and soil fertility^9–11^.

Dung beetle taxa differ in their nesting techniques and are classified as dwellers, tunnelers, or rollers^12^, which have a major impact on ecological functions, such as dung removal efficiency^13^. Many of the beetle species excavate the soil in distinct ways, with varying diameters and sizes, resulting in different micro-environments with different GHG fluxes^14,15^ and enhanced nutrient cycling by transferring soil carbon more efficiently and favouring bacterial soil diversity^12,16,17^.

Therefore, different dung beetle species were applied individually or in combination with dung used to fertilize pearl millet [*Pennisetum glaucum* (L.)], where N_2_O flux and NH_3_ volatilization and forage productivity were evaluated. Thus, we hypothesized that the presence of dung beetles would reduce N_2_O emission and NH_3_ volatilization and would increase the crop yield due to the enhanced N cycling and reduced N losses.

## Results

**Figure 1:**
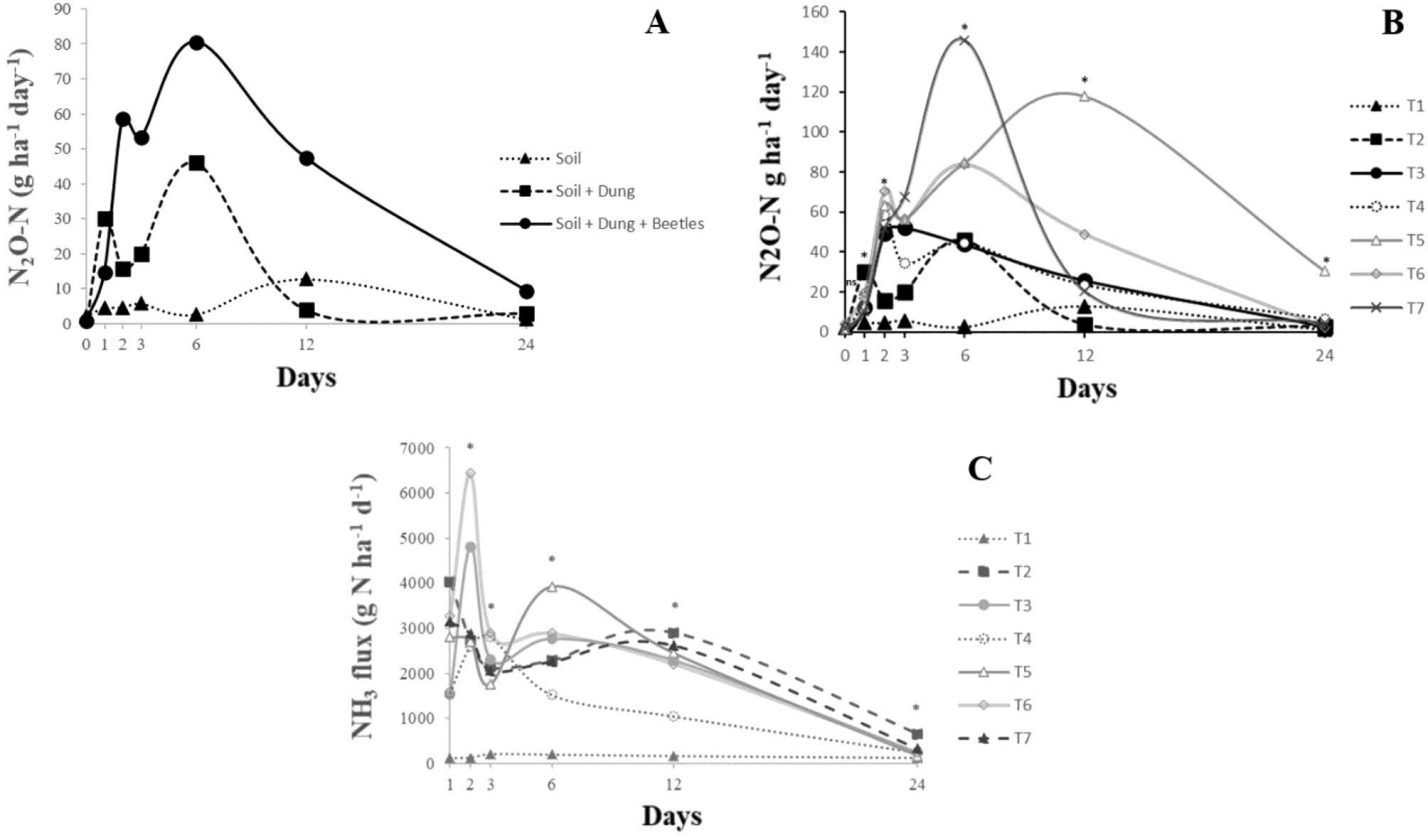
Nitrous oxide flux and ammonia volatilization from soil, soil + dung, and soil + dung + dung beetles over a 24-d period. (**A**) Nitrous oxide flux average of all dung beetle treatments vs. their control, (**B**) nitrous oxide flux over time from livestock dung under contrasting dung beetle species, (**C**) ammonia flux over time. T1: just soil, T2: soil + dung, T3: soil + dung + *O. taurus* (OT), T4: soil + dung + *D. gazella* (DG), T5: soil + dung + *P. vindex* (PV), T6: soil + dung + OT + DG, T7: soil + dung + OT + DG + PV. Asterisk: indicates significant difference at the 0.05 probability level among treatments in the same month, according to orthogonal contrast test.

There was a sampling day × treatment interaction on fluxes of N_2_O (*P* < 0.001), with average emissions ranging from 2 g N_2_O-N ha^-1^ day^−1^ for all treatments in day 0 after treatment application and 80 g N_2_O-N ha^−1^ day^−1^ on day 6 to bucket with just dung and bucket with dung + dung beetle species, respectively (Fig. 1A). The fluxes of N_2_O from bucket with dung beetle species (the group of all species and *P. vindex* alone) were the greatest and differed significantly to bucket with dung and bucket with just soil over time (*P* < 0.001), except in day 2, when N_2_O flux was greater to just dung than dung + beetles. Dung beetle activity increased N_2_O-N flux by 71% and 79% when compared to bucket with dung and bucket with just soil on day 2, respectively. The increase occurred mainly on days 1, 2, and 6 (Fig. 1A).

Fluxes of N_2_O were greater over time for dung + beetles than for soil and soil + dung (*P* < 0.001). Moreover, *O. taurus* and *D. gazella* had the least N_2_O emission compared to other beetle species over time (Fig. 1B). The N_2_O flux from bucket with dung (T2) increased over time, but decreased from 45 to 2.9 g N_2_O-N ha^−1^ day^−1^ from day 6 to 24. The T3 and T4 demonstrated lesser N_2_O-N emission among the dung beetle treatments in days 0, 1, and 12, averaging of −3, 12.3, 25.8 and −1, 17.5, 23.5 g N_2_O-N ha^−1^ day^−1^, respectively. Treatment 7 presented the greatest peak of N_2_O-N (145.7 g N_2_O-N ha^−1^ day^−1^) on day 6, while in day 12 the N_2_O did not differ from T3, and T4 (Fig. 1B). Treatment 1 bucket had the least N_2_O flux average (1.09 g N_2_O-N ha^−1^ day^−1^). Treatment 5 showed a progressive increase over time, with the greatest peak of N_2_O-N on days 12 and 24.

There was a sampling day × treatment interaction (*P* < 0.05) on ammonia volatilization, which varied from 6431 g NH_3_-N ha^−1^ for T6 on day 2 to 241 g NH_3_-N ha^−1^ on day 24. Treatments T3 and T4 presented the least averages with 1536 and 1575 g of NH_3_-N ha^−1^, respectively, when compared to other treatments (Fig. 1C). Treatment T4 presented less volatilization of NH_3_-N on days 6, 12, and 24 with averages of 1526, 1048, and 245 g ha^−1^ when compared to other treatments. The T5 showed a peak on day 6, which was greater (*P* < *0.*001) than T1, T2, T3, and T4. The T1 presented the least NH_3_-N emission, and it did not significantly vary over time (Fig. 1C).

**Figure 2:**
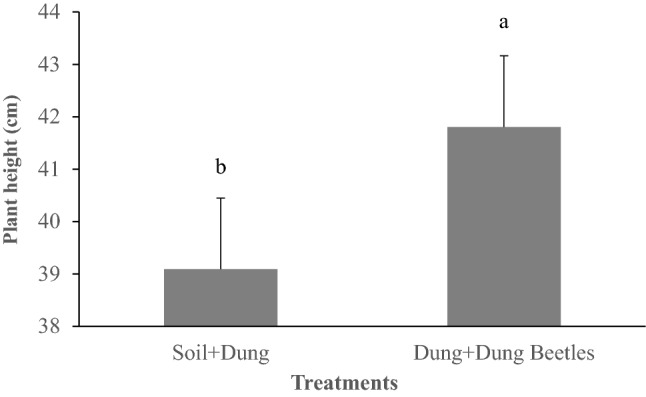
Pearl millet height in the presence or absence of dung beetles. Asterisk: means followed by different letters are significantly different between treatments, according to polynomial contrasts.

All treatments with dung beetle species resulted in taller pear millet plants (*P* < 0.05) in treatments where dung was applied . Pearl millet plants cultivated in the presence of dung beetles were 41.8 cm tall, which was greater than millet plants that was cultivated in absence of beetles (39.9 cm; P = 0.035; Fig. 2). Results of dung removal, soil nitrogen concentration, pear millet forage production, nitrogen yield and PCA analyses are demonstrated in Supplementary Figs. S1, S2, S3 and S4 online).

**Figure 3:**
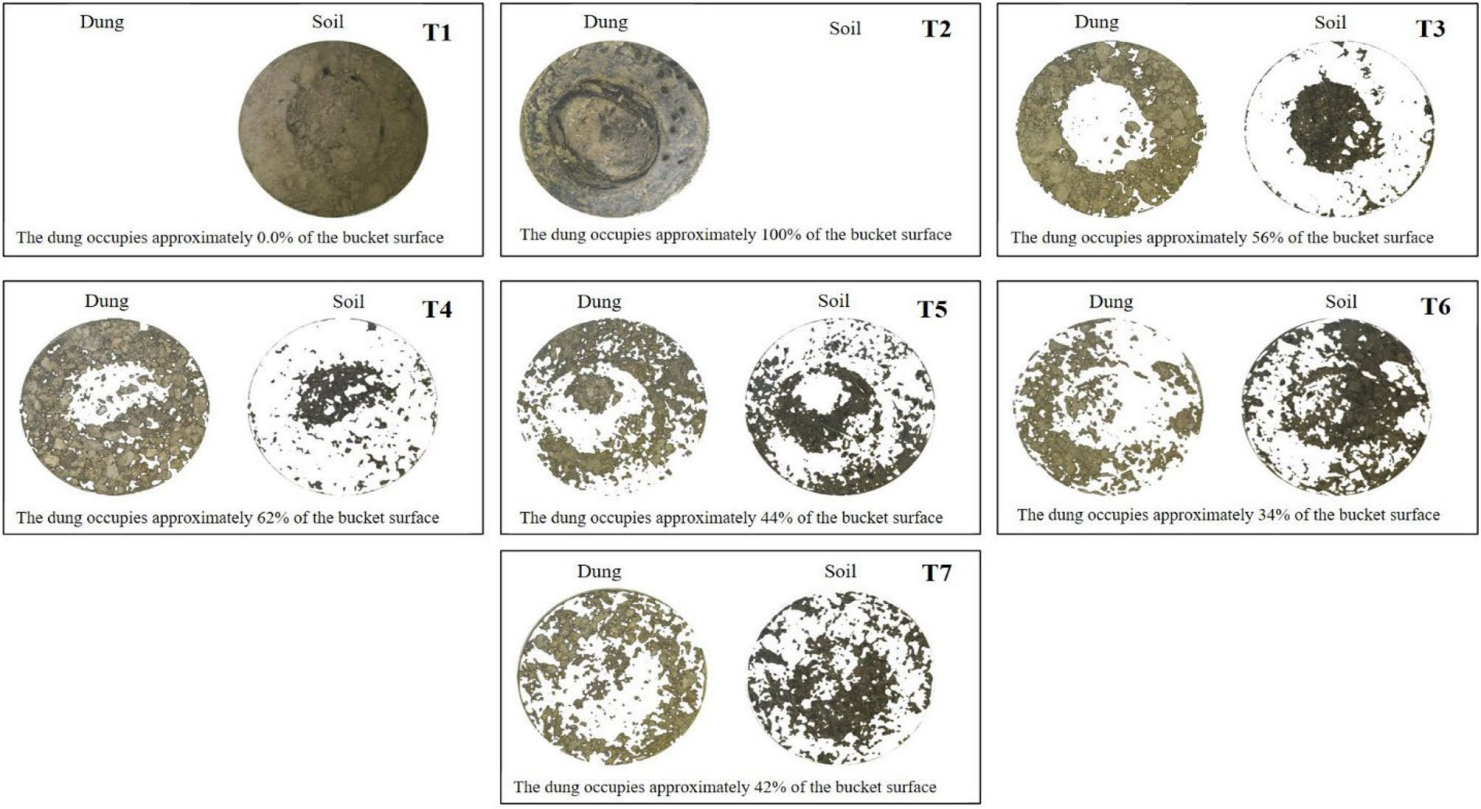
Dung removal of single dung beetle species and their combinations. T1: just soil, T2: soil + dung, T3: soil + dung + *O. taurus* (OT), T4: soil + dung + *D. gazella* (DG), T5: soil + dung + P. vindex (PV), T6: soil + dung + OT + DG, T7: soil + dung + OT + DG + PV.

Dung removal efficiency by beetles is showed on Fig. 3. The smallest species *O. taurus* and *D. gazella* had the highest proportions of dung on the bucket surface (T3 = 56% and T4 = 62%, respectively), while the largest species *P. vindex* had the smallest area occupied by dung (T5 = 44%). Additionally, the combinations of species 1 + 2 and 1 + 2 + 3 occupied a smaller surface area of the buckets with dung (T6 = 34% and T7 = 42%, respectively). This indicates the efficacy of dung beetles in dung removal, with a lower proportion of dung on the surface of the bucket and a higher proportion of dung buried in the soil.

## Discussion

Previous research has found that dung beetle activity increased N_2_O emissions from cow dung deposited in tropical regions with greatest fluxes on days 15, 20, and 30, after dung application^17^, which also have been suggested to increase NO_3_^−^ levels by aerating the substrate^18^. Nonetheless, the N_2_O dynamics during denitrification may also be related to soil depth, labile organic carbon, soil nitrate, and microbial biomass C^19^. Another possibility to increased N_2_O emission is because dung beetle made balls with the cow dung, maintaining it at a high moisture and favoring an anaerobic condition, greater N concentration, and more available carbon. These are perfect conditions for N_2_O emission to occur, providing optimal conditions for denitrifying bacteria^20^.

The NH_3_-N emission is dependent of soil pH, moisture, texture, cation exchange capacity (CEC), and soil temperature, as well as the wind speed and air temperature^21^. In the current study, N_2_O-N and NH_3_-N showed the greatest peaks in the presence of dung beetles. Soil temperature and humidity averaged 27 °C and 37% in the soil-only buckets, and 26 °C and 78% in the buckets with dung + beetles, respectively. This reduction of NH_3_ emission is explained by the formation of a superficial crust on the dung. This crust can act as a physical barrier to the wind, preventing NH_3_ volatilization^22^. Furthermore, NH_3_ tends to diffuse between fecal matter, in which it will be converted into NH_4_+, making NH_3_ emission even more difficult^23^.

The diversity of dung beetles varies within the seasons, and their activities and effects related to dung decomposition are likely to differ by species^24^. This fact likely has an impact not only on dung decomposition but also on N_2_O emissions^25^ and NH_3_ volatilization. In this study, *P. vindex* increased N losses even when mixed with other species. This is probably because *P. vindex* has longer lifetime (can live over a year) than the other species^26^. Furthermore, *P. vindex* presents in its gut 24% of bacteria that belong to *Enterecoccace amilyliy* (Scheleifer and Kilpper-Bälz, 1984)^27^, which could contribute to denitrifying process as *Enterococcus casseliflavus* (Collins, 1984)^28^. Evans et al.^29^ demonstrated that dung beetle affected N_2_O flux during the late summer season by modifying the moisture-dependent gas transport processes. Reduced N_2_O emission from all treatments with dung in the current study in the first day of evaluation might be because organic N needs to go through several processes before returning to atmosphere as N_2_O^30^.

*D. gazella* is characterized by its high dispersal ability^31,32^ and broad tolerance to climatic conditions^33^, being an effective competitor and invader, and reducing the population of other beetle species in a specific assemblage^34^. On the other hand, small dung beetles remove more dung, due to shorter legs and heads that helps to bury and to make holes^35,36^. This could explain why *D. gazella* combining with *O. taurus* from this study removed more dung than the other singles species. Furthermore, when *D. gazella* was grouped with *P. vindex*, the dung removal was reduced, but still more efficient than alone (single species).

More than 85 of N consumed by cattle returns to the soil via excreta^37^. Dung beetle activity could bury and mineralize fecal N in a short period of time, transforming the organic N and P into an inorganic form available to the plant^38^. This might have increased soil N concentration (see Supplementary Fig. S2), resulting in increasing N_2_O emission because more N is available as a substrate to denitrifying bacteria. Despite several studies have shown that dung beetles increase N_2_O emission^6,14,35^, others have shown their important role in the soil nutrient cycling, increasing the soil organic matter by 159 g in 600 m^2^ (equivalent to 2647 kg ha^−1^)^18^. Although we did not measure organic matter content, images of each bucket demonstrated that dung beetles removed and buried dung from the soil surface. This could promote the action of soil microbial respiration and affect the decomposition rate of soil organic matter^39^.

Dung has N and other nutrients required for plant development, which can improve tillering and increase forage mass^40,41^. However, in the current study, the lower herbage accumulation in the second harvest for all treatments might have been due to low soil nutrient availability since the first harvest extracted the major remaining nitrogen incorporated by dung decomposition.

Our findings suggest that dung beetle activity of this study may speed up nitrogen mineralization from applied dung. According to Badenhorst et al.^42^, nutrient concentration in the vegetation increases significantly where dung beetles were active. In this study, the activity of dung beetles did not affect the pearl millet N concentration for any of the treatments. The plant herbage accumulation has strong correlation with plant height due to the meristem level growth, which is associated with the production of new cells and initiation of new organs^43^. This corroborates our findings due to positive correlation of pear millet biomass and its height. Furthermore, the PCA just revealed the strong effect of dung beetles to soil nitrogen and greenhouse gases, through a positive correlation between them (see Supplementary Fig. S4 online).

## Conclusions

The presence of dung beetles in dung from cattle provided important ecosystem services by improving nutrient cycling and increasing retention of soil nitrogen. Greater soil N resulted in greater plant biomass and N concentration. Dung beetles, however, provided some disservices due to increasing nitrogen losses from cattle dung instead of reducing it. *D. gazella* tended to reduce the total N losses as N_2_O and NH_3_ from dung and it was more efficient in the removal of dung from the bucket soil surface when combining with *O. taurus*, which enhances nutrient cycling in a grassland.

## Materials and methods

All procedures involving animals were conducted in accordance with the guidelines and regulations from Institutional Animal Care and Use Committee (IACUC) of the University of Florida (protocol #201509019). Tis manuscript is reported in accordance with ARRIVE guidelines.

### Site description

This study was carried out at the North Florida Research and Education Center, in Marianna, FL (30°46′35″N 85°14′17″W, 51 m.a.s.l). The trial was performed in two experimental years (2019 and 2020) in a greenhouse.

The soil used was collected from a pasture of rhizoma peanut (*Arachis glabrata* Benth.) and Argentine bahiagrass (*Paspalum notatum* Flügge) as the main forages. Without plant and root material, only soil was placed into buckets, as described below in the bucket assemblage section. Soil was classified as Orangeburg loamy sand (fine-loamy-kaolinitic, thermic Typic Kandiudults), with a pH_water_ of 6.7, Mehlich-1-extratable P, K, Mg and Ca concentrations of 41, 59, 63, 368 mg kg^−1^, respectively. Average of minimum and maximum daily temperature and relative humidity in the greenhouse for September and November (September for beetle trial due seasonal appearance of beetles, and October and November to the Pear Millet trial) in 2019 and 2020 were 11 and 33 °C, 81%; 10 and 35 °C, 77%, respectively.

### Biological material determination

**Figure 4:**
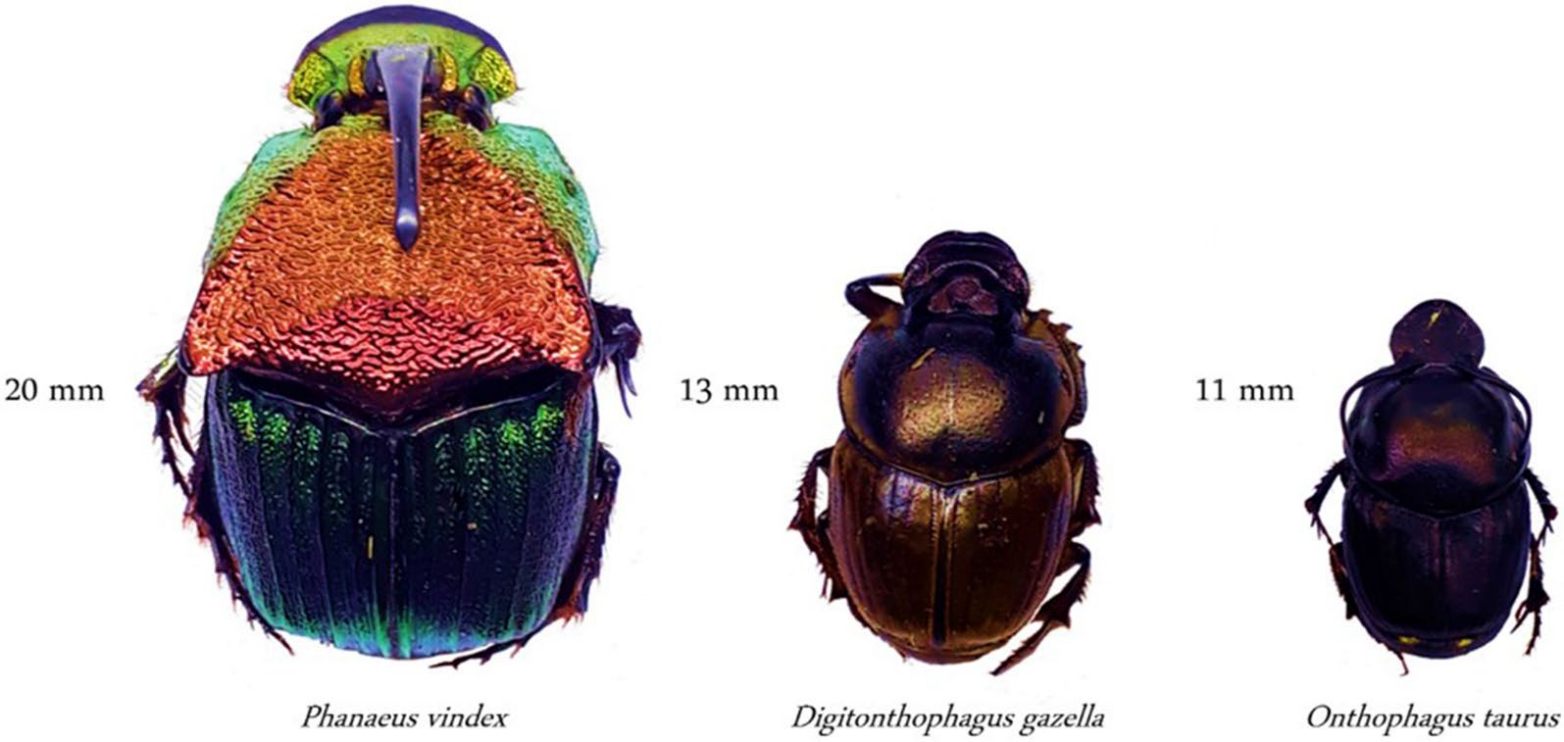
Most abundant dung beetle species in Marianna, FL used in the current study. Credits: Carlos C.V. García.

To select the species of beetles, a previous dung beetle sampling was performed in the grazing experiment in the same area (grass and legume forage mixture) to determine the number of dung beetle species according to the functional groups as described by Conover et al.^44^^.^ Beetles were pre-sampled from March 2017 to June 2018, where Tunnelers group were dominant and represented by *Onthophagus taurus* (Schreber), *Digitonthophagus gazella* (Fabricius), *Phanaeus vindex* (MacLeay), *Onthophagus oklahomensis* (Brown), and *Euniticellus intermedius* (Reiche). Other species were present but not abundant, including *Aphodius psudolividus* (Linnaeus)*, Aphodius carolinus* (Linnaeus), and *Canthon pilularius* (Linnaeus) identified as Dweller and Roller groups, respectively. The pre-sampling indicated three species from the Tunneler group were more abundant, and thereby, were chosen to compose the experimental treatments (Fig. 4).

### Beetles collection and experimental treatments

Three species of common communal dung beetles were used: *O. taurus* (1), *D. gazella* (2), and *P. vindex* (3). Treatments included two treatments containing only soil and soil + dung without beetles were considered as Control 1 (T1) and Control 2 (T2), respectively. Isolated species T3 = 1, T4 = 2, T5 = 3 and their combinations T6 = 1 + 2 and T7 = 1 + 2 + 3. Dung beetles were trapped in the pasture with grazing animals using the standard cattle-dung-baited pitfall traps, as described by Bertone et al.^41^. To avoid losing samples due to cattle trampling, 18 traps were randomized in nine paddocks (two traps per paddock) and installed protected by metal cages, and after a 24-h period, beetles were collected, and the traps removed. Table 1 shows the number of dung beetles, their total mass (used to standardize treatments) per treatment, and the average mass per species. To keep uniformity across treatments we kept beetle biomass constant across species at roughly 1.7 to 1.8 g per assemblage (Table 1). Twenty-four hours after retrieving the beetles from the field traps, they were separated using an insect rearing cage, classified, and thereafter stored in small glass bottles provided with a stopper and linked to a mesh to keep the ventilation and maintaining the beetles alive.

**Table 1:** Total number and biomass of dung beetles per treatment.

Treatment	*Ot*	*Dg*	*Pv*	Total mass (g)
1	–	–	–	–
2	–	–	–	–
3	43	–	–	1.72
4	–	30	–	1.75
5	–	–	7	1.82
6	25	13	–	1.76
7	9	6	4	1.75

Dung beetle species	Average mass (mg)/beetle			
*Phanaeus vindex* (Pv)	261.15			
*Digitonthophagus gazella* (Dg)	58.57			
*Onthophagus taurus* (Ot)	40.12			

### Buckets assemblage

**Figure 5:**
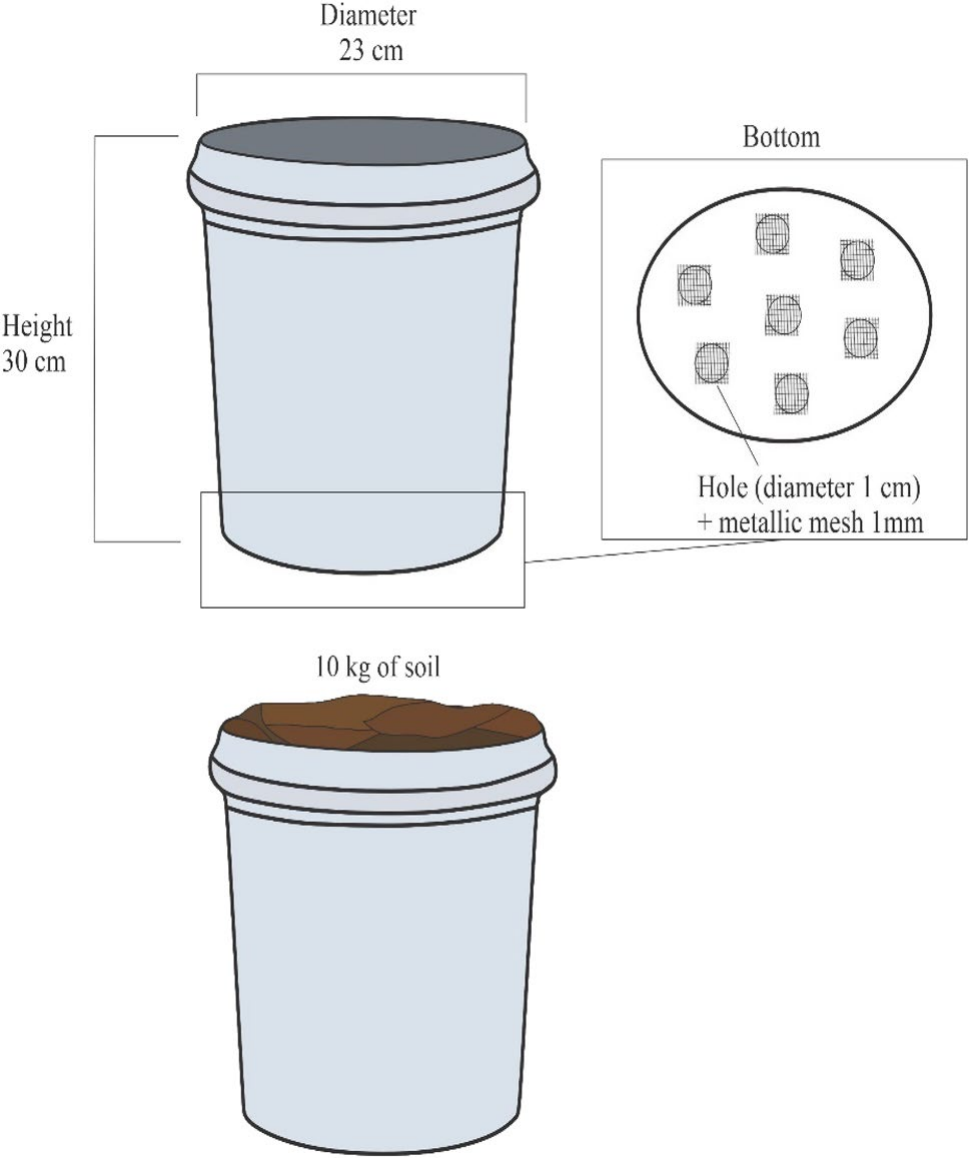
Bucket plastic bucket details for dung beetle trial.

The soil used in the buckets was collected from the grazing trial in two experimental years (August 2019 and August 2020) across nine paddocks (0.9 ha each). The 21 plastic buckets had a 23-cm diameter and 30-cm (0.034 m^2^) and each received 10 kg of soil (Fig. 5). At the bottom of the recipient, seven holes were made for water drainage using a metallic mesh with 1-mm diameter above the surface of the holes to prevent dung beetles from escaping. Water was added every four days to maintain the natural soil conditions at 60% of the soil (i.e., bucket) field capacity (measured with the soil weight and water holding capacity of the soil). Because soil from the three paddocks had a slightly different texture (sandy clay and sandy clay loam), we used them as the blocking factor.

The fresh dung amount used in the trial was determined based on the average area covered by dung and dung weight (0.05 to 0.09 m^2^ and 1.5 to 2.7 kg) from cattle in grazing systems, as suggested by Carpinelli et al.^45^. Fresh dung was collected from Angus steers grazing warm-season grass (bahiagrass) pastures and stored in fridge for 24 h, prior to start the experiment. A total of 16.2 kg of fresh dung was collected, in which 0.9 kg were used in each bucket. After the dung application, dung beetles were added to the bucket. To prevent dung beetles from escaping, a mobile plastic mesh with 0.5 mm diameter was placed covering the buckets before and after each evaluation. The experiment lasted for 24 days in each experimental year (2019 and 2020), with average temperature 28 °C and relative humidity of 79%, acquired information from the Florida Automated Weather Network (FAWN).

### Chamber measurements

**Figure 6:**
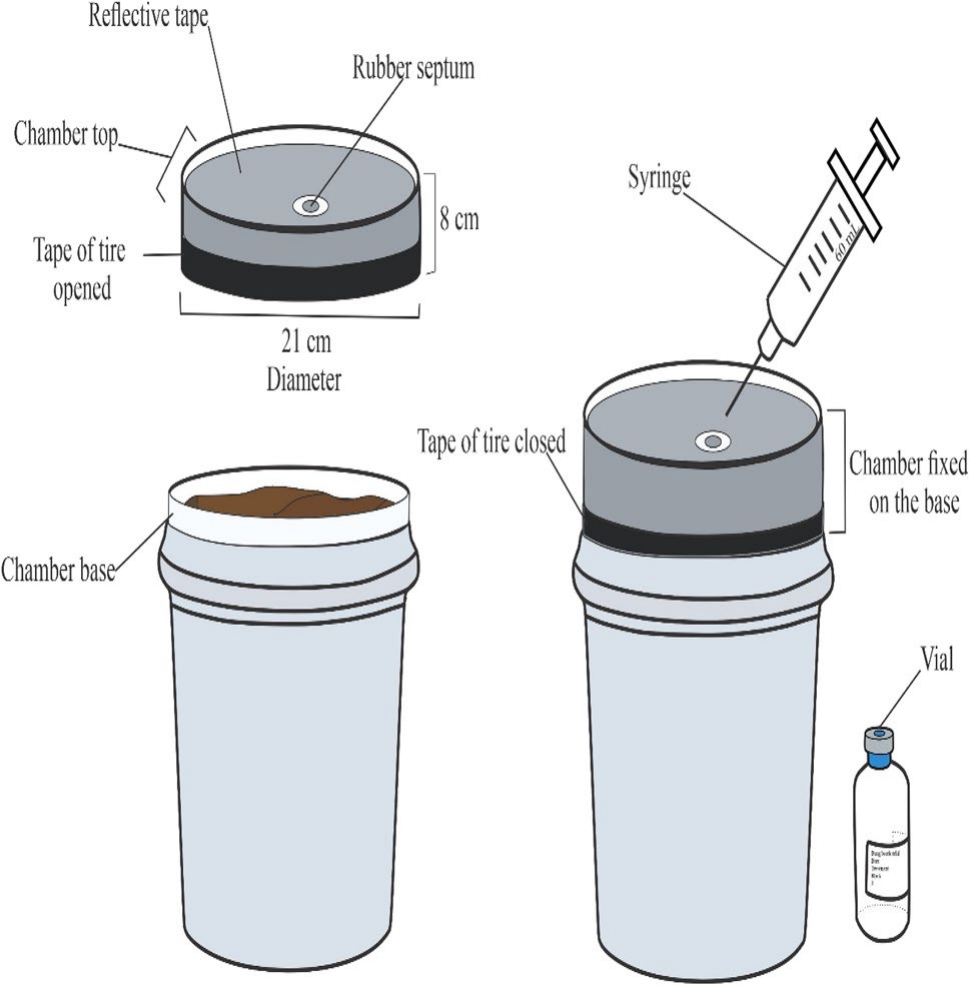
Static chamber details and instruments for GHG collection in the dung beetle trial.

The gas fluxes from treatments were evaluated using the static chamber technique^46^. The chambers were circular, with a radius of 10.5 cm (0.034 m^2^). Chamber bases and lids were made of polyvinyl chloride (PVC), and the lid were lined with an acrylic sheet to avoid any reactions of gases of interest with chamber material (Fig. 6). The chamber lids were covered with reflective tape to provide insulation, and equipped with a rubber septum for sampling^47^. The lid was fitted with a 6-mm diameter, 10-cm length copper venting tube to ensure adequate air pressure inside the chamber during measurements, considering an average wind speed of 1.7 m s^−1^^48,49^. During measurements, chamber lids and bases were kept sealed by fitting bicycle tire inner tubes tightly over the area separating the lid and the base. Bases of chambers were installed on top of the buckets to an 8-cm depth, with 5 cm extending above ground level. Bases were removed in the last evaluation day (24th) of each experimental year.

### Gas fluxes measurements

The gas fluxes were measured at 1000 h following sampling recommendations by Parkin & Venterea^50^, on seven occasions from August 28th to September 22nd in both years (2019 and 2020), being days 0, 1, 2, 3, 6, 12, and 24 after dung application. For each chamber, gas samples were taken using a 60-mL syringe at 15-min intervals (t0, t15, and t30). The gas was immediately flushed into pre-evacuated 30-mL glass vials equipped with a butyl rubber stopper sealed with an aluminium septum (this procedure was made twice per vial and per collection time). Time zero (t0) represented the gas collected out of the buckets (before closing the chamber). Immediately thereafter, the bucket lid was tightly closed by fitting the lid to the base with the bicycle inner tube, followed by the next sample deployment times.

Gas sample analyses were conducted using a gas chromatograph (Trace 1310 Gas Chromatograph, Thermo Scientific, Waltham, MA). For N_2_O, an electron capture detector (350 °C) and a capillary column (J&W GC packed column in stainless steel tubing, length 6.56 ft (2 M), 1/8 in. OD, 2 mm ID, Hayesep D packing, mesh size 80/100, pre-conditioned, Agilent Technologies) were used. Temperature of the injector and columns were 80 and 200 °C, respectively. Daily flux of N_2_O-N (g ha^−1^ day^−1^) was calculated as described in Eq. (1):

1 $${\text{F}}\, = \,{\text{A}}*{\text{dC}}/{\text{dt}}$$

where F is flux of N_2_O (g ha^−1^ day^−1^), A is the area of the chamber, and dC/dt is the change of concentration in time calculated using a linear method of integration by Venterea et al.^49^.

### Ammonia volatilization measurement

**Figure 7:**
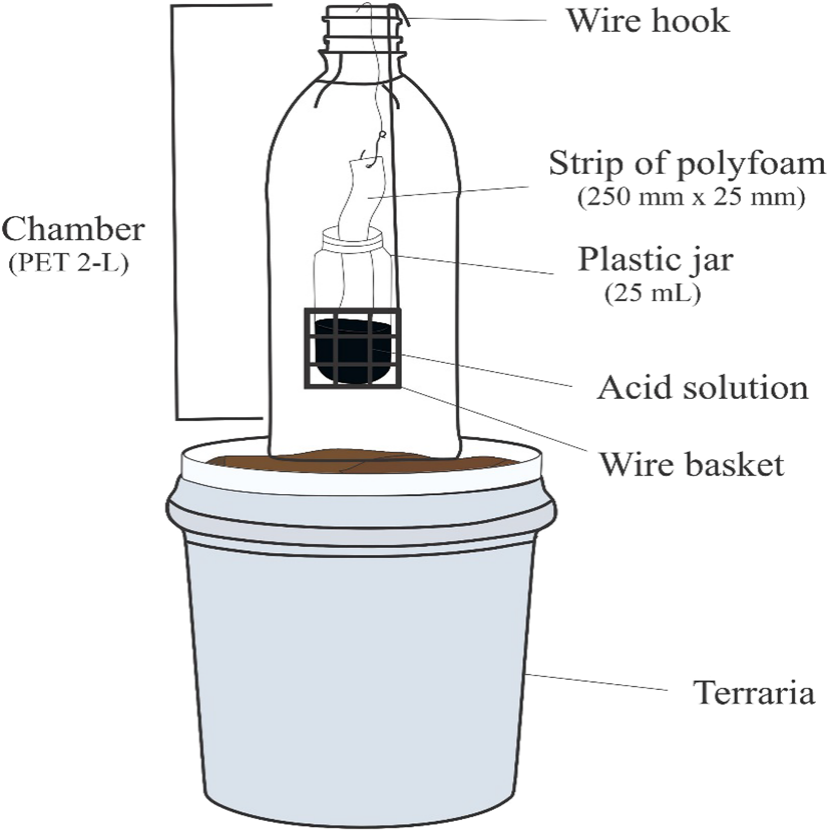
Mobile ammonia chamber details for ammonia measurement in dung beetle trial. Adapted from Araújo et al.^51^.

Ammonia volatilization was measured using the open chamber technique, as described by Araújo et al.^51^. The ammonia chamber was made of a 2-L volume polyethylene terephthalate (PET) bottle. The bottom of the bottle was removed and used as a cap above the top opening to keep the environment controlled, free of insects and other sources of contamination. An iron wire was used to support the plastic jar. A strip of polyfoam (250 mm in length, 25 mm wide, and 3 mm thick) was soaked in 20 ml of acid solution (H_2_SO_4_ 1 mol dm^−3^ + glycerine 2% v/v) and fastened to the top, with the bottom end of the foam remaining inside the plastic jar. Inside each chamber there was a 250-mm long wire designed with a hook to support it from the top of the bottle, and wire basket at the bottom end to support a plastic jar (25 mL) that contained the acid solution to keep the foam strip moist during sampling periods (Fig. 7). The ammonia chambers were placed installed in the bucket located in the middle of each experimental block after the last gas sampling of the day and removed before the start of the next gas sampling.

### Nutrient cycling

Photographs of the soil and dung portion of each bucket were taken twenty-four hours after the last day of gas flux measurement sampling to determine the dung removal from single beetle species and their combination. In the section on statistical analysis, the programming and statistical procedures are described. After this procedure, seeds of pearl millet were planted in each bucket. After 5 days of seed germination plants were thinned, maintaining four plants per bucket. Additionally, plants were clipped twice in a five-week interval, with the first cut occurring on October 23rd and the second cut occurring on November 24th, in both experimental years. Before each harvest, plant height was measured twice in the last week. In the harvest day all plants were clipped 10 cm above the ground level. Samples were dried at 55 °C in a forced-air oven until constant weight and ball-milled using a Mixer Mill MM 400 (Retsch, Newton, PA, USA) for 9 min at 25 Hz, and analyzed for total N concentration using a C, H, N, and S analyzer by the Dumas dry combustion method (Vario Micro Cube; Elementar, Hanau, Germany).

### Statistical analysis

Treatments were distributed in a randomized complete block design (RCBD), with three replications. Data were analyzed using the Mixed Procedure from SAS (ver. 9.4., SAS Inst., Cary, NC) and LSMEANS compared using PDIFF adjusted by the *t*-test (*P* < 0.05). Gas sampling day and harvest were used as repeated measures. Treatments were considered the fixed effect, while block and experimental year were considered random effects. Polynomial contrasts were used to test the effect of dung absence (Soil *vs.* Soil + Dung), the effect of dung beetle (Soil + Dung *vs.* Soil + Dung + Dung beetle species) and the effect of each species and their combinations (Species 1 *vs.* 2, 3, 1 + 2, 1 + 2 + 3) on N_2_O emission and nutrient cycling. A principal component analysis (PCA) was used to o get a better understanding about the effect of dung beetle treatments, using a biplot graph with the PRCOMP functions from the stats package (R Core Team)^52^ and a ggbiplot from the ggbiplot package^53^, with the assistance of the vegan, tidyverse and devtools packages^54^.

The following steps were taken to determine the proportions of interest, "soil" and "dung," after segmenting the original images for the exclusive analysis of the portion corresponding to the contents of the buckets: conversion of colored images to grayscale images using the RGB model^55^; smoothing boundary transitions using defocusing and the Multidimensional Gaussian Filter technique^56^; Manual thresholding involves analyzing the histogram of grayscale images to generate binary images and determining the proportion of "soil" and "dung" in the binary images by counting the pixels of interest. The Python programming language^57^, as well as the imageio^58^ numpy^59^ matplotlib^60^ , and scikit-image libraries^61^, were used to analyze the images and create the figures that resulted from the process.

## Supplementary Information

Supplementary Information.

## Data Availability

The datasets used and/or analyzed during the current study available from the corresponding author on reasonable request.
