# Supplementary material for: The role of dung beetle species in nitrous oxide emission, ammonia volatilization, and nutrient cycling
Source: Sci Rep. 2023 Mar 2;13:3572. doi: 10.1038/s41598-023-30523-0 (PMC9981724; doi:10.1038/s41598-023-30523-0)
Supplement: Supplementary file 1 — Supplementary Information. [file 41598_2023_30523_MOESM1_ESM.docx]

# SUPPLEMENTARY MATERIALS

# Soil nitrogen content

There was a treatment effect (*P*<0.05) on soil nitrogen content. The two control treatments (soil and soil + dung) did not differ (*P>*0.05). The soil from pot with dung + beetle application (treatment 3, 4, 5, 6 and 7) presented greater nitrogen concentration compared with treatment 1 and 2 (Fig. S1).

**Fig. S1.** Effect of feces and dung beetle species on soil nitrogen concentration. T1: just soil, T2: soil + dung, T3: soil + dung + *O. taurus* (OT), T4: soil + dung + *D. gazella* (DG), T5: soil + dung + *P. vindex* (PV), T6: soil + dung + OT+DG, T7: soil + dung + OT+DG+PV. Different letters in lowercase indicate statistically significant differences among treatments, according to student test.

# Herbage accumulation

There was a harvest × treatment interaction (*P<*0.05) on HA of pear millet. Dung application had a positive effect on the HA. The greatest HA was observed in all treatments with dung application in the first harvest with average of 8 g of DM pot^-1^ (pot area of 0.32 m^2^), greater (*P*<0.05) than T1 (control with just soil), which averaged 5 g of DM pot^-1^. In the second harvest, treatments did not differ among them,presenting the least DM values (Fig. S2).

**Fig. S2.** Pearl millet herbage accumulation (HA) comparing the two controls and different dung beetle species effect. T1: just soil, T2: soil + dung, T3: soil + dung + *O. taurus* (OT), T4: soil + dung + *D. gazella* (DG), T5: soil + dung + *P. vindex* (PV), T6: soil + dung + OT+DG, T7: soil + dung + OT+DG+PV. Different letters in lowercase indicate statistically significant differences among treatments, according to orthogonal contrast.

# Nitrogen yield

There was a treatment effect (*P*<0.05) on N yield (Fig. S3). Treatments T3, T6, and T7 resulted in greater N yield for the pearl millet than T1, T2, T4, and T5. The T3, T6, and T7 also had more soil N available, with average of 0.34, 0.30, and 0.31 g N pot^-1^, respectively.

**Fig. S3.** Pearl millet nitrogen yield comparing the dung and dung beetle effect.

T1: just soil, T2: soil + dung, T3: soil + dung + *O. taurus* (OT), T4: soil + dung + *D. gazella* (DG), T5: soil + dung + *P. vindex* (PV), T6: soil + dung + OT+DG, T7: soil + dung + OT+DG+PV. Different letters in lowercase indicate statistically significant differences among treatments, according to orthogonal contrast test.

# Principal component analysis

The first and second principal component (PC1, PC2) explained 29.5% and 21.4% of the variability in the data set, respectively. The PC1 provided the highest variation, and HA had a positive correlation with plant height (PH). Treatment 3 (*O. taurus*) showed more relationship with soil nitrogen (SN) and NY. Treatment 5 (*P. vindex*) had a positive association with nitrous oxide emission (N_2_O), as well the Treatment 2 and other beetle treatments, but a wide variation, and that is confirmed in the greater fluxes of N_2_O when *P. vindex* was present (Figure 1, main text). Treatment 1 did not have dung nor beetles and that might explain its negative correlation with all the variables. From all beetle treatments, Treatment 4 was least associated with N_2_O. Both PC explained 50.9% of the variability in the data set; this percentage is not enough to provide a better description (Fig. S4).


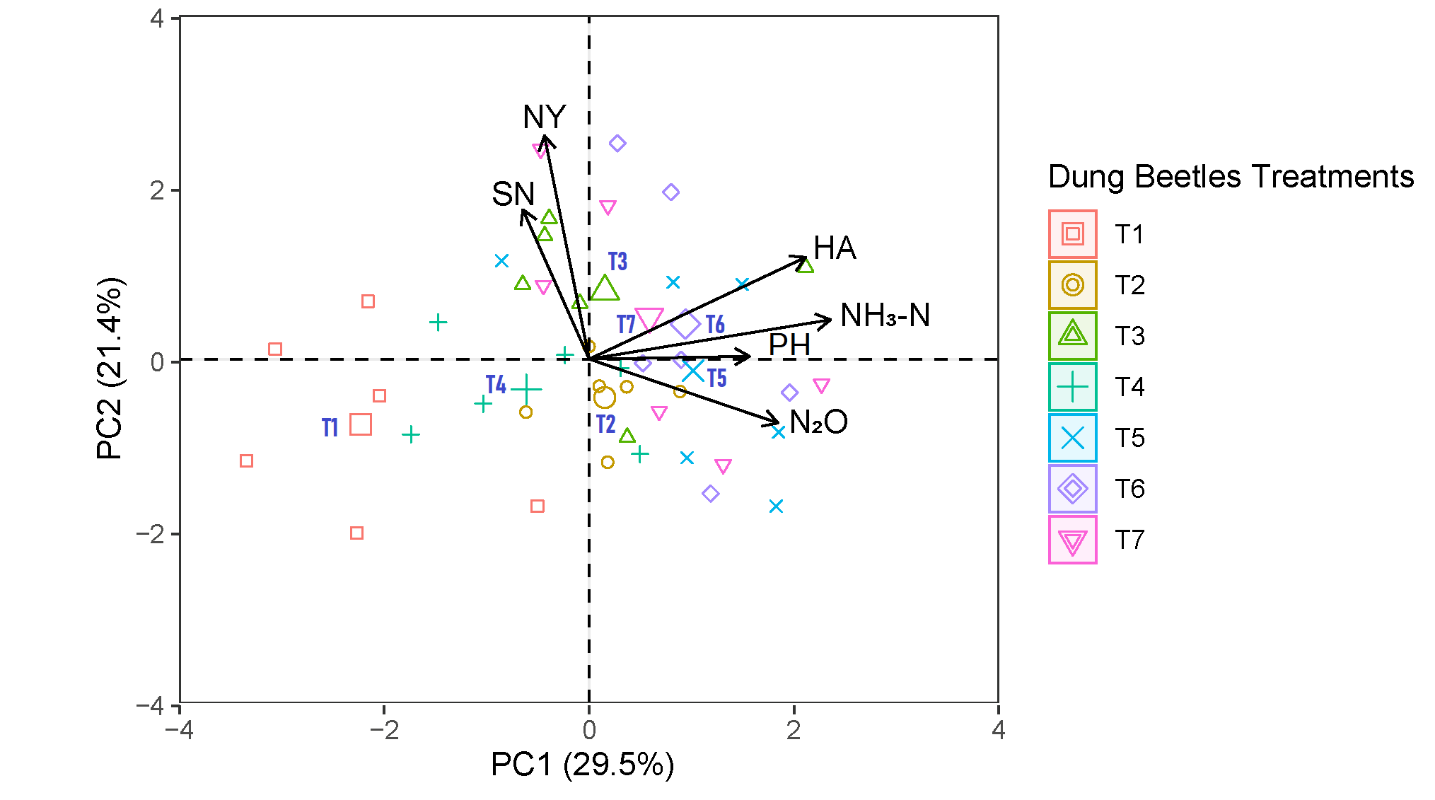


**Fig. S4.** Principal component analysis of nitrogen emissions and nutrient cycling variables of dung beetle experiment in NFREC, Marianna, FL. T1: just soil, T2: soil + dung, T3: soil + dung + *O. taurus* (OT), T4: soil + dung + *D. gazella* (DG), T5: soil + dung + *P. vindex* (PV), T6: soil + dung + OT+DG, T7: soil + dung + OT+DG+PV. NY: nitrogen yield, SN: soil nitrogen, HA: herbage accumulation of pear millet, NH_3_-N: ammonia volatilization, PH: plant height, N_2_O: nitrous oxide emission.

# Aditional information about beetles combination

*Onthophagus taurus* (1), *Digitonthophagus gazella* (2), and *Phanaeus vindex* (3)were the species used. That design was used because we tested all of the combinations with the most abundant species, with species 1 and 2 being the first and second most abundant, respectively. The goal was to see if increasing diversity aids in the degradation of cow manure. Also, this information was added as supplementary material.
